
JOURNAL INFORMATION
==============================
NLM Title Abbreviation: Curr Med Sci
Journal ID: Curr Med Sci
NLM Journal ID: 101729993

Current Medical Science

ISSN: 2096-5230
EISSN: 2523-899X

ARTICLE INFORMATION
==============================
PMCID: PMC9800345
PMID: 36580238
DOI: 10.1007/s11596-022-2660-y
Article ID: 2660
Article version: 1
Subjects: Erratum

Erratum to: TOPK Activation Exerts Protective Effects on Cisplatin-induced Acute Kidney Injury

Zhang Hui 1449457465@qq.com

Dong Qing-qing
Shu Hua-pan
Tu Yu-chi
Liao Qian-qian
Yao Li-jun drylj@hotmail.com

grid.33199.31 0000 0004 0368 7223 Department of Nephrology, Union Hospital, Tongji Medical College, Huazhong University of Science and Technology, Wuhan, 430022 China
Electronic publication date: 2022 Dec 29
Print publication date: 2022
Volume: 42
Issue: 6
First page: 1334
Last page: 1334
Copyright: © The Author(s) 2022
License: Open Access This article is licensed under a Creative Commons Attribution 4.0 International License https://creativecommons.org/licenses/by/4.0/), which permits use, sharing, adaptation, distribution and reproduction in any medium or format, as long as you give appropriate credit to the original author(s) and the source, provide a link to the Creative Commons licence, and indicate if changes were made. The images or other third party material in this article are included in the article’s Creative Commons licence, unless indicated otherwise in a credit line to the material. If material is not included in the article’s Creative Commons licence and your intended use is not permitted by statutory regulation or exceeds the permitted use, you will need to obtain permission directly from the copyright holder. To view a copy of this licence, visit http://creativecommons.org/licenses/by/4.0/.
License URL: https://creativecommons.org/licenses/by/4.0/

Erratum for: TOPK Activation Exerts Protective Effects on Cisplatin-induced Acute Kidney Injury. 9 6 2022 Curr Med Sci 10.1007/s11596-022-2545-0 35678915
issue-copyright-statement: © Huazhong University of Science and Technology 2022

==============================
The online version of the original article can be found at 10.1007/s11596-022-2545-0

